# Multigeneration toxicity of imidacloprid and thiacloprid to *Folsomia candida*

**DOI:** 10.1007/s10646-017-1765-8

**Published:** 2017-01-23

**Authors:** Cornelis A.M. van Gestel, Claudia de Lima e Silva, Thao Lam, Jacco C. Koekkoek, Marja H. Lamoree, Rudo A. Verweij

**Affiliations:** 1 Department of Ecological Science, Faculty of Earth and Life Sciences, Vrije Universiteit, De Boelelaan 1085, Amsterdam, 1081 HV The Netherlands; 2 Department of Environment and Health, Faculty of Earth and Life Sciences, Vrije Universiteit, De Boelelaan 1085, Amsterdam, 1081 HV The Netherlands

**Keywords:** Neonicotinoid insecticides, Soil ecotoxicity, Reproduction

## Abstract

In a recent study, we showed that the springtail *Folsomia candida* was quite sensitive the neonicotinoid insecticides imidacloprid and thiacloprid. This study aimed at determining the toxicity of both compounds to *F. candida* following exposure over three generations, in natural LUFA 2.2 standard soil. In the first generation, imidacloprid was more toxic than thiacloprid, with LC_50_s of 0.44 and 9.0 mg/kg dry soil, respectively and EC_50_s of 0.29 and 1.5 mg/kg dry soil, respectively. The higher LC_50_/EC_50_ ratio suggests that thiacloprid has more effects on reproduction, while imidacloprid shows lethal toxicity to the springtails. In the multigeneration tests, using soil spiked at the start of the first generation exposures, imidacloprid had a consistent effect on survival and reproduction in all three generations, with LC_50_s and EC_50_s of 0.21–0.44 and 0.12–0.29 mg/kg dry soil, respectively, while thiacloprid-exposed animals showed clear recovery in the second and third generations (LC_50_ and EC_50_ > 3.33 mg/kg dry soil). The latter finding is in agreement with the persistence of imidacloprid and the fast degradation of thiacloprid in the test soil.

## Introduction

Neonicotinoids are widely used to protect crops against herbivorous insects, with application as seed-dressing agents (Tomizawa and Casida 2003; Douglas and Tooker 2015), soil treatment and spraying (Goulson 2013; Van der Sluijs et al. 2015). Neonicotinoids are systemic, being distributed throughout the plants via the sap stream, in this way making the entire plant toxic to, i.e., the target insects. They specifically bind to nicotinergic acetylcholine receptors (nAChR) on the post-synaptic membrane of the neurons of insects. They compete with ACh neurotransmitters to bind to and activate the nAChR, an effect called agonistic binding. The irreversible binding leads to excessive ion flows (Na^+^, K^+^, Ca^2+^) through cellular membranes and prolonged action potentials, causing overexcitement of the neurons. Exposed animals show signs of disorientation and paralysis, from which they eventually die (Buckingham et al. 1997; Goulson 2013; Matsuda et al. 2001; Millar and Denholm 2007; Sheets 2001; Tomizawa and Casida 2003).

Neonicotinoids are divided into three groups, *N*-nitroguanidines, *N*-cyanoguanidines, and nitromethylenes (Goulson 2013), which differ in toxicity, with the *N*-cyanoguanidines being less toxic than the *N*-nitroguanidines (Iwasa et al. 2004). The *N*-nitroguanidine imidacloprid was about 800 times more toxic to honeybees upon acute dermal exposure than the related *N*-cyanoguanidine thiacloprid (Iwasa et al. 2004). Shi et al. (2011) showed that one of the target organisms, the aphid *Aphis gossypii*, was about 7.5 times more sensitive to imidacloprid than to thiacloprid when exposed dermally for 48 h. The fact that both insecticides are usually applied at similar dosages (Pisa et al. 2015), however, suggests they are equally effective against target organisms. This indicates that the difference in toxicity in short-term laboratory tests does not translate to differences in longer-term efficacy in the field. What the reason is for this discrepancy remains unclear.

Following application more than 90% of the neonicotinoid dose may stay in the soil or may reach the soil by washing off the treated crop, where the compounds may persist and accumulate, potentially threatening non-target soil organisms (Laurent and Rathahao 2003; Goulson 2013). Persistence in soil may contribute to the exposure to these compounds, causing potential long-term effects on soil organisms. Thiacloprid is a factor of 10 less persistent in soil than imidacloprid, with half-lives of 3.4–74 and 28–1250 days, respectively found in laboratory tests (Goulson 2013; Bonmatin et al. 2015). EFSA (2008) concluded that the laboratory half-life for imidacloprid degradation in soil was 106 to 293 days while (under European conditions) the field half-lives were 40–288 days. Imidacloprid may therefore persist in soil and cause adverse effects on multiple generations of soil organisms, especially of species with short life cycles. For thiacloprid, long-term exposure may occur when the compound is sprayed frequently, like in fruit-growing and horticulture.

Relatively little is known about the effects of neonicotinoids in the soil (EASAC 2015, Van der Sluijs et al. 2015). Recently, we determined the toxicity of imidacloprid and thiacloprid to five different species of soil invertebrate. In 21–28 day exposures, imidacloprid generally was more toxic than thiacloprid, and springtails (*Folsomia candida*) were most sensitive (de Lima e Silva et al. 2017). This study however, did not assess long-term consequences of exposure the neonicotinoids, over multiple generations.

Springtails are highly abundant (Ponge et al. 1997) and of major importance for the functioning of the soil ecosystem (Thimm et al. 1998). They contribute to fragmentation of dead organic material, stimulating its degradation by microorganisms and therefore stimulating nutrient cycling (Hanlon 1981; Elkins and Whitford 1982; Seastedt 1984). For that reason, springtails have been adopted as standard test species in soil ecotoxicology by OECD (2009) and ISO (1999).

This paper aims at further exploring the toxicity of imidacloprid and thiacloprid to the springtail *F. candida* by examining their long-term multigeneration response. Exposure over multiple generations may reveal possible effects because of the accumulation of damage due to long-term toxicant stress, while it might also reveal the potential for springtails to adapt to these compounds. By exposing the animals to soil spiked only once, also the potential for recovery (Ernst et al. 2016) is included in this assessment. It was hypothesized that toxicity of the more stable imidacloprid would persist over different generations, while that of the faster degradable thiacloprid would decrease with following generations.

## Materials and methods

### Test organisms

*F. candida* were taken from cultures at the Department of Ecological Science at the Vrije Universiteit in Amsterdam. To obtain age-synchronized animals, adults from the culture were separated in boxes with a layer of plaster of Paris over a period of 2–3 days to lay eggs, after which they were removed. After removal of the adults, the eggs were incubated under a 12 h light/12 h dark regime at 20 °C and 75% relative humidity, where they hatched and grew. Juveniles of 10–12 days old were used for testing.

### Test soil and chemicals

All tests were performed in the natural standard LUFA 2.2 (Lufa Speyer, Germany), a loamy sand with 1.59 ± 0.13% organic carbon, pH in 0.01 M CaCl_2_ of 5.4 ± 0.2, and water holding capacity (WHC) of 43.5 ± 2.8% of its dry weight.

Pure imidacloprid and thiacloprid (purity 98%) were kindly provided by Bayer CropScience, Monheim, Germany. Test concentrations of imidacloprid and thiacloprid were based on the results of earlier tests. Stock solution of both compounds in water were prepared, to spike the chemicals into the soil and at the same time bringing moisture content to the desired level of 50% of the WHC. To ensure complete thiacloprid dissolution, a small amount of acetone was added to the stock solution. This stock solution was used to spike soil with the highest test concentration and further diluted for spiking soil with the lower concentrations tested. Considering the low amount of acetone needed to make thiacloprid completely dissolve, no attempts were made to correct for that by adding acetone to the lower concentrations and control in the multigeneration test. All soils were thoroughly mixed to achieve a homogenous distribution of the test chemicals in the test soil.

### Toxicity tests—general principles

Tests were performed in 100 ml glass jars, containing c. 30 g moist soil, using five replicate test jars for each exposure concentration. Each jar received ten 10–12 day old animals from age-synchronized cultures, after checking their health under a binocular microscope. The animals were fed with a few grains of dry baker’s yeast (Instant yeast from Algist Bruggeman N.V, Ghent, Belgium) weekly, in small amounts to avoid fungal growth. All incubations took place at 20 ± 2 °C at 75% Relative Humidity and a photoperiod of 16:8 dark: light hours. Test jars were weighed at the start, so that water loss could be monitored on a weekly basis and replenished with deionized water if needed. After exposure, all animals were extracted by flotation and pictures were taken to enable counting the number of surviving adults and juveniles produced using the software package ImageJ with a Cell Counter extension.

### Multigeneration test

Multigeneration tests started with a control, 7 imidacloprid (0, 0.001, 0.003, 0.01, 0.03, 0.1, 0.3, 1.0 mg/kg dry soil) and 9 thiacloprid concentrations (0, 0.0015, 0.0045, 0.014, 0.041, 0.11, 0.31, 1.1, 3.3, 10.0 mg/kg dry soil). Batches of soil sufficient to perform three toxicity tests were prepared. After filling test jars for the parent generation exposure, the remaining spiked soil was stored in glass jars in a climate room at 20 °C for subsequent exposure of the next two generations. Moisture content of the spiked soil was checked regularly by weighing the jars, and replenished by adding deionized water if needed.

After 28 days (35 days for the second generation), the juveniles from the same exposure concentration were pooled in the same container with a moist plaster of Paris bottom and incubated overnight before being transferred to the next cycle of exposure. This experiment spanned a total of three continuous generations (referred to as F0, F1, and F2).

Soil samples for chemical analysis of imidacloprid and thiacloprid in the test soil were taken at the start of the exposure of each new generation.

### Reference chemical

In addition to the neoninotinoid tests, a toxicity test was performed with a reference compound to confirm the sensitivity of the test system. Springtails were exposed to soil spiked with boric acid solutions (diluted from 99.5% pure boric acid (H_3_BO_3_) from Sigma-Aldrich, USA) to obtain concentrations of 0, 20, 40, 80, 160, 320 mg/kg dry soil.

### Chemical analysis

The analytical method to determine actual imidacloprid and thiacloprid concentrations in the test soils followed the principles of the QuEChERS extraction method modified from Anastasiades et al. (2003) and Payá et al. (2007). Briefly, the procedure used was as follows: approx. 1 g moist soil was taken for low concentration treatments (<1 mg/kg), and 50 µL internal standard (Imidacloprid-d4 and Thiacloprid-d4, 250 µg/L) was added. The sample was shaken for 20 min at 250 rpm with 2 mL methanol and centrifuged for 3 min at 1500 rpm. The methanol phase was passed through a granular Na_2_SO_4_ column, evaporated till dryness and the residue taken up in 3 mL acetonitrile. For high concentration samples (>1 mg/kg), the soil was first shaken with methanol and centrifuged. Subsequently an aliquot of 100 µL was taken and together with 50 µL internal standard (Imidacloprid-d4 and Thiacloprid-d4, 250 µg/L) added to 1 mL methanol, passed through a granular Na_2_SO_4_ column, evaporated to dryness and taken up in 3 mL acetonitrile. For clean-up, the acetonitrile extract was added to a tube filled with Supel QuE (C_18_ 150 mg, PSA 150 mg, MgSO_4_ 900 mg, Sigma Aldrich), shaken by hand for 2 min and centrifuged for 3 min at 3000 rpm. The supernatant was evaporated till dryness under gentle nitrogen stream and the residue taken up in 200 µL of a water-methanol (9/1) mixture.

Extracts were analyzed using LC-MS and quantified by isotope dilution (imidacloprid-d4 and thiacloprid-d4). The neonicotinoids were separated on a 100 × 2.1 mm, 2.6 µm Kinetex XB-C_18_ column (Phenomenex), applying a gradient of 5 mM ammonium formate pH 4 buffer and methanol, at a flow rate of 0.3 mL/min. The LC system (Agilent 1200 series) was coupled to a 6410 triple quadrupole MS (Agilent) using electrospray ionization (ESI) with data acquisition in positive ion mode. The transitions for imidacloprid were 256.1 > 175/209 (quantifier/qualifier) and for imidacloprid-d4 260 > 179. For thiacloprid the transitions were253.1 > 126/90 (quantifier/qualifier) with 257 > 126 for thiacloprid-d4.

### Data analysis

Using data on the imidacloprid and thiacloprid concentrations measured at different times after spiking the soil in the multigeneration test, half-life (DT50) values were estimated assuming first order degradation kinetics. The equation C = C_0_ * e^−k*t^ was fitted to the data, with *C* = concentration at time *t* in days, C_0_ is concentration at *t* = 0 and *k* = degradation rate constant (day^−1^). DT50 was derived as ln(2) / k.

LC_50_ (lethal concentration killing 50% of the test organisms), EC_50_ and EC_10_ (effect concentrations causing 50 and 10% reduction of the number of juveniles) were estimated with a logistic dose response model (Haanstra et al. 1985). If no proper fit was obtained, LC_50_ values were calculated using the trimmed Spearman-Karber method (Hamilton et al. 1977/78). No observed effect concentrations (NOEC) were determined applying a one-way ANOVA followed by a one-sided Dunnett’s post hoc test at a significance level of *p* < 0.05. All statistical analysis, except for the Trimmed Spearman Karber method, were run in SPSS 23.

## Results

### Chemical analysis

Table S1 shows the concentrations measured in soil samples taken from the multigeneration tests. Unfortunately, samples from *t* = 0 for thiacloprid got lost and the samples analysed were taken on 28 (F1) and 63 (F2) days after the start of the test. For imidacloprid, the samples analysed were taken from the first (start of exposure of F0 generation) and the 28th (start of F1 generation) day of the experiment. Unfortunately, due to time constraints it was not possible to analyse samples for imidacloprid taken after 63 days. For imidacloprid, recovery was 77.6–89.9% of the nominal concentration, which further declined to 68.1–83.8% after 28 days of incubation. These decline rates were too small to estimate half-lives for the degradation of imidacloprid. For thiacloprid, concentrations measured after 28 days were 91.2–101% for the two lowest concentrations analysed (1.1 and 3.3 mg/kg dry soil), and 56.7% at 10 mg/kg. After 63 days of incubation, thiacloprid, concentrations had decreased to 7.6–22.0% of the nominal values with highest loss recorded at the highest test concentration. Samples of the two lowest thiacloprid concentrations tested (0.0137 and 0.113 mg/kg) were only analysed after 63 days, and contained 22.0 and 19.8% of the nominal concentration (Table S1). From these data, DT50 values of 10–12 days were estimated for the degradation of thiacloprid.

### Multigeneration tests

Control performance in the multigeneration tests is summarized in Table S2. In the tests with imidacloprid, control mortality was rather high and increased with increasing generation, while juvenile numbers decreased and coefficient of variation increased with following generations. For the imidacloprid controls, test validity criteria set by ISO (1999) and OECD (2009) (adult mortality < 20%, mean number of juveniles > 100 per jar; coefficient of variation < 30%) were only met for juvenile numbers and coefficient of variation in the first two generation, while for thiacloprid all tests were valid.

**Fig. 1:**
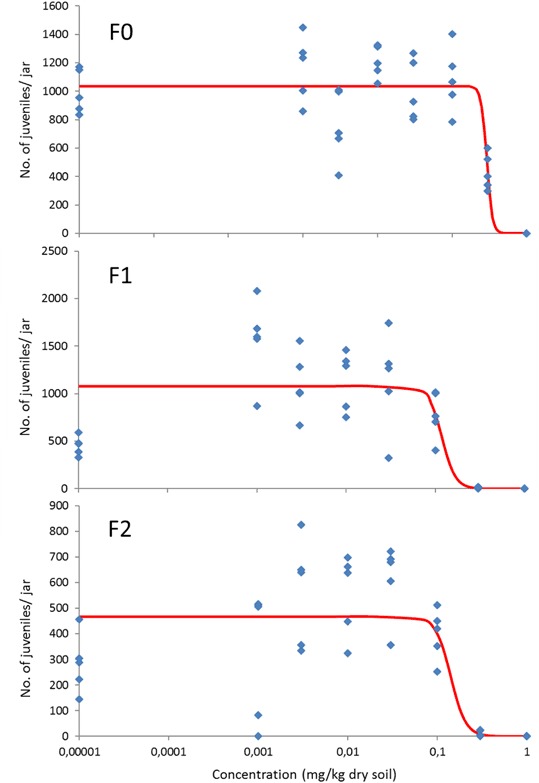
Dose-response relationships for the effect of imidacloprid on the reproduction of *Folsomia candida* exposed for three consecutive generations to LUFA 2.2 soil spiked at the start of the experiment. Concentrations are nominal values at the start of the test. The control is set at a low value of 0.00001 mg/kg dry soil. Points are measured values, lines show the fit of a logistic dose-response model to the data

*Imidacloprid* did cause a consistent and significant dose-related decrease of the survival of *F. candida* in all three generations (Figure S1 in the Supporting Information). Estimated LC_50_s non-significantly decreased from 0.44 mg/kg dry soil for the parent generation to 0.39 and 0.21 mg/kg dry soil for the F1 and F2 generations, respectively (Table 1). Reproduction was dose-related decreased by imidacloprid in all three generations, with EC_50_s for the F0, F1, and F2 generations being 0.29, 0.12, and 0.14 mg/kg dry soil, respectively and EC_10_s declining from 0.24 to 0.080 and 0.098 mg/kg dry soil, respectively (Table 1). It was not possible to estimate 95% confidence intervals for the EC_50_ and EC_10_ due to the large variation in the data and the very steep dose-response curves (Fig. 1). Although in the F1 and F2 generations control reproduction was much lower than at the lowest test concentrations, we did include the controls when calculating EC_50_ and EC_10_ values. The NOEC for effects on the F0, F1, and F2 generations was 0.1 mg/kg dry soil. This value probably is less robust due to the low reproduction in the controls of the latter two generations.

**Table 1:** LC_50_, EC_50_, and NOEC values for the multigenerational toxicity of imidacloprid and thiacloprid to the springtail *Folsomia candida* in LUFA 2.2 soil

Compound	Generation	LC_50_ (mg/kg dry soil)	EC_50_ (mg/kg dry soil)	EC_10_ (mg/kg dry soil)	NOEC (mg/kg dry soil)
Imidacloprid	F0	0.44	0.29	0.24	0.10
		(0.27–0.72)	(−)	(−)	
	F1	0.39	0.12	0.080	0.10
		(0.31–0.50)	(−)	(−)	
	F2	0.21	0.14	0.098	0.10
		(0.14–0.30)	(−)	(−)	
Thiacloprid	F0	9.0	1.5	0.23	0.11
		(5.6–14)	(0.70–2.3)	(−)	
	F1	>3.3	>3.3	>3.3	>3.3
	F2	>3.3	>3.3	>3.3	3.3
Boric acid		127 (115–141)	51 (47–54)	29 (25–33)	20

Animals were exposed for three consecutive generations to soil spiked with these compounds at the start of the experiment. Also shown are 95% confidence intervals for the LC_50_ and EC_50_ values where calculable. All values are based on nominal concentrations at the start of the exposures. Also included are data on the toxicity of boric acid, which was tested as a reference compound

In the F0 generation, both adult mortality and reproduction of the springtails were dose-related reduced by *thiacloprid* with estimated LC_50_, EC_50_ and EC_10_ values of 9.0, 1.5 and 0.23 mg/kg dry soil, respectively (Table 1; Figures S2 and 2). At the highest concentration tested (10 mg/kg dry soil), only few juveniles (on average 3 per jar) were produced, not allowing to start an F1 exposure. At the second highest concentration tested (3.3 mg/kg dry soil), reproduction of the F0 generation was significantly reduced, but no effects on juvenile numbers were seen in the F1 and F2 generations. LC_50_ and EC_50_ values therefore are >3.3 mg/kg dry soil, while NOEC was ≥3.3 and 1.1 mg/kg dry soil for the F1 and F2 generations, respectively (Table 1).

**Fig. 2:**
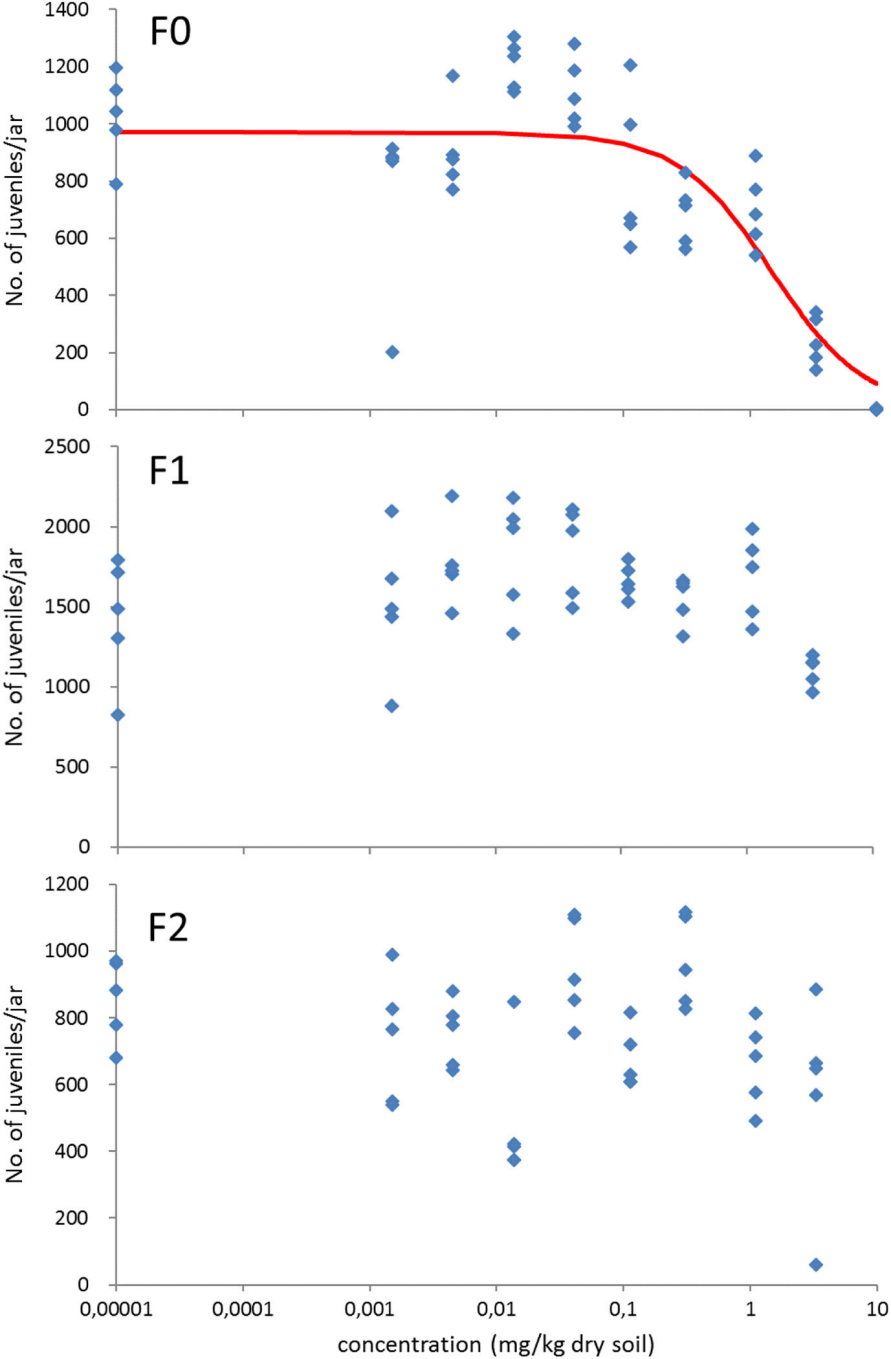
Dose-response relationships for the effect of thiacloprid on the reproduction of *Folsomia candida* exposed for three consecutive generations to LUFA 2.2 soil spiked at the start of the experiment. Concentrations are nominal values at the start of the test. The control is set at a low value of 0.00001 mg/kg dry soil. Points are measured values, the line shows the fit of a logistic dose-response model to the data

### Boric acid

The test with the reference compound showed fairly high control mortality, but good control reproduction. Boric acid dose-related reduced both survival and reproduction, with estimated LC_50_, EC_50_ and EC_10_ values of 127, 51 and 29 mg/kg dry soil, respectively, while NOEC was 20 mg/kg dry soil (Table 1; Figure S3).

## Discussion

Imidacloprid was more toxic to springtails than thiacloprid. The effects of imidacloprid upon long-term exposure over three generations were persistent while for thiacloprid there is potential for recovery.

### Control performance and toxicity of the reference chemical

Not all tests performed were valid, because of either a too low control survival and/or a too high variation in the number of juveniles. But even when control survival was much lower than the required 80%, juvenile numbers produced per jar by far exceeded the required number of 100 in all tests (Table S2). This suggests that the animals were healthy and the low survival did not affect the outcome of the reproduction tests. In some cases reproduction (imidacloprid F1 and F2; Fig. 1) in the controls was much lower than at the lower treatment levels. Nevertheless, in all cases consistent dose-related response relationships were found. Reproducibility of the tests was high with good agreement of LC_50_ and EC_50_ values among the different tests as well as with earlier tests performed in our laboratory (see below).

In the multigeneration test, the low control performance and large variation in the F1 and F2 generation may be due to the transfer of rather small juveniles (Campiche et al. 2007), which may also be from different clutches introducing bigger differences in starting age. This may also explain the fairly large scatter in the data, a phenomenon also seen in the data of Leon Paumen et al. (2008) who did a similar multigeneration toxicity test exposing *F. candida* to phenanthrene. For that reason, Ernst et al. (2016) recommended an intermediate phase of 2 weeks in between the exposures of different generations, which may reduce variability and enhance validity of the test.

The reference chemical boric acid was more toxic than expected, with an EC_50_ of 51 mg/kg dry soil. This is a factor of 2 lower than the value of 100 mg/kg dry soil for OECD artificial soil, mentioned in the test guideline (OECD 2009). Taking into account that the LUFA 2.2 soil used has a lower OM content (3 vs. 5% in artificial soil) and also lower pH (5.4 vs. 6.0), the sensitivity of *F. candida* test population seems not to deviate much from expected.

### Toxicity of both compound and comparison with literature data

The LC_50_ and EC_50_ values for the toxicity of imidacloprid found in this study are within the range set by previous studies in our lab (Table S3). The values reported by Idinger (2002) for the toxicity of imidacloprid in the commercial formulation Confidor 70WP, tested in artificial soil, also are in the same range. Some LC_50_s reported in the literature, however, are somewhat higher, which could be explained by several factors. The high LC_50_ of 21 mg/kg dry soil reported by Alves et al. (2014) may be explained from the short test duration (14 days compared to 28 days in this study), the use of a different soil type (tropical artificial soil) with a much higher OM content (10%) and the use of a commercial formulation (Gaucho 600FS) instead of the pure active substance. Idinger (2002) found a 14-d LC_50_ of 2.6 mg/kg for imidacloprid in the commercial formulation Confidor 70WG, also tested in an artificial soil. The use of an artificial soil with higher OM contents probably also explains the somewhat higher LC_50_ and EC_50_ values reported by Reynolds (2008). EFSA (2008) concluded on an NOEC for imidacloprid toxicity to *F. candida* of 1.25 mg/kg dry soil, which is higher than the value of 0.1 mg/kg found in this study. For two commercial formulations, EFSA (2008) found lower NOECs of 0.2–0.32 mg a.s./kg. Laboratory conditions such as temperature and light/dark regime may affect the metabolism of test animals, leading to differential toxicity of imidacloprid. This may also explain the higher EC_50_ (>1 mg/kg) found by Alves et al. (2014), who performed their study at a temperature of 25 °C (compared to 20 °C in our study).

LC_50_ and EC_50_ values for the toxicity of thiacloprid were in good agreement with those found in an earlier study in our lab and the ones reported by Akeju (2014) (Table S3).

### Multigeneration effects

In the multigeneration test, the toxicity of imidacloprid remained constant across the three generations tested, which is in accordance with the reported soil half-lives, which range from 106 to 293 days (EFSA 2008), and the concentrations measured in the test soil (Table S1). The concentration decrease measured in our study suggests that half-life was ≥125 days, which also agrees with the values mentioned by EFSA (2008). The constancy of imidacloprid toxicity also suggests that the animals were not able to recover from exposure, nor that they were able to develop resistance against the test compound. The latter probably will require a much longer exposure, over many generations.

For thiacloprid, the F0 generation showed high mortality and produced only few juveniles at 10 mg/kg dry soil. Therefore no exposure of F1 and F2 generations to this concentration was possible. The LC_50_ for effects on survival of the F1 and F2 generations was higher than the highest test concentration remaining (3.3 mg/kg dry soil), and at this concentration springtail reproduction of the F1 and F2 generations was affected by less than 50% (Fig. 2). The recovery of the springtail populations suggests that the toxic strength of thiacloprid decreased with every generation of the test animals. This is confirmed by the chemical analysis showing that the concentration at the 10 mg/kg treatment already was reduced to approx. 56% of the nominal one after 28 days. At the lower test concentrations, still 90–100% of the compound was present after 28 days, but at all treatment levels thiacloprid concentrations subsequently decreased with a half-life of 10–12 days. This finding agrees with literature data reporting half-lives for the degradation of thiacloprid in soil between 3.4 and 74 days (Goulson 2013; Bonmatin et al. 2015), and field-based half-lives of 9–27 days (European Commission 2004).

The data on trans-generational effect were confounded by several other factors, including the fact that the animals went through several transfers during flotation and extraction, which might have damaged some animals and/or affected their overall fitness. During transfer, all juveniles of the same exposure concentration were pooled in the same box with a plaster of Paris bottom and stored overnight before being transferred to the next round of exposure the next day. This discontinuity of exposure, although not significantly long, may have affected their sensitivity.

Only few multigeneration toxicity tests on springtails have been reported. Leon Paumen et al. (2008) determined the effect of phenanthrene on 10 consecutive generations of *F. candida* in soil that was freshly spiked before the start of each new generation, to ensure more or less constant exposure. They found significant effects on survival and reproduction in the first four generations. Our experiment with imidacloprid gave a similar result: all three generations showed a constancy in toxicity for survival and reproduction after being exposed to imidacloprid over three generations, which can be explained from the high persistency of this compound. In case of thiacloprid, however, recovery was seen as a consequence of its fast degradation. Taking into account the degradation of the test compound and the potential for recovery of the test organisms in multigeneration exposures was the reason why Ernst et al. (2016) advocated spiking soil only once. This procedure aimed at simulating a more realistic single peak exposure of a plant protection product to *F. candida* following population recovery in the next generation after aging and/or degradation of the compounds in soil.

## Electronic supplementary material

Supplementary Information
