# Supplementary material for: Multigeneration toxicity of imidacloprid and thiacloprid to Folsomia candida
Source: Ecotoxicology. 2017 Jan 23;26(3):320–8. doi: 10.1007/s10646-017-1765-8 (PMC5397431; doi:10.1007/s10646-017-1765-8)
Supplement: Supplementary file 1 — Supplementary Information [file 10646_2017_1765_MOESM1_ESM.docx]

Supporting Information to

**Multigeneration toxicity of imidacloprid and thiacloprid to *Folsomia candida***

Cornelis A.M. van Gestel^1^*, Claudia de Lima e Silva^1^, Thao Lam^1^, Jitske M. Brouwer^1^, Jacco C. Koekkoek^2^, Marja H. Lamoree^2^, Rudo A. Verweij^1^

^1^Department of Ecological Science, Faculty of Earth and Life Sciences, Vrije Universiteit, De Boelelaan 1085, 1081 HV Amsterdam, The Netherlands

^2^Institute of Environmental Studies, Faculty of Earth and Life Sciences, Vrije Universiteit, De Boelelaan 1085, 1081 HV Amsterdam, The Netherlands

*Corresponding author

e-mail: [kees.van.gestel@vu.nl](mailto:kees.van.gestel@vu.nl)

tel. +31-20-5987079

**Table S1.** Concentrations of imidacloprid and thiacloprid measured in LUFA 2.2 soil used in the multi-generation toxicity test with *Folsomia candida*. The soil was freshly spiked, incubated in a climate room at 20 ºC, and analyzed after 28 and 63 days (at the start and end of the second generation exposures). All values are mean ± SD (n=3) in mg/kg dry soil; n.a. not analyzed.

| Imidacloprid | | | thiacloprid | | |
| --- | --- | --- | --- | --- | --- |
| nominal | T=0 | T=28 d | nominal | T=28 | T=63 d |
| 0 | 0.00013±0.00022 | n.a. | 0 | <0.05 | n.a. |
| 0.01 | 0.0078±0.0028 | n.a. | 0.014 | n.a. | 0.003±0.0002 |
| 0.1 | 0.082±0.0018 | 0.068±0.0025 | 0.113 | n.a. | 0.022±0.00044 |
| 0.3 | 0.27±0.014 | 0.23±0.0092 | 1.11 | 1.1±0.35 | 0.12±0.0054 |
| 1.0 | 0.86±0.022 | 0.84±0.083 | 3.33 | 3.0±0.42 | 0.26±0.0038 |
|  |  |  | 10 | 5.7±0.27 | 0.76±0.015 |

**Table S2.** Performance of the *Folsomia candida* controls of the multi-generation toxicity tests with imidacloprid and thiacloprid in LUFA 2.2 soil. Animals were exposed for 28 days, except for the imidacloprid F1 where exposure was 35 days. Also included are controls the test with boric acid, which was used as a reference chemical. Values printed in bold do not meet the validity criteria set by the test guidelines (ISO 1999; OECD 2009).

| Compound | Generation | Mean adult mortality (%) | Mean no. of juveniles/ test jar | Coefficient of variation (%) |
| --- | --- | --- | --- | --- |
| Imidacloprid | F0 | **34** | 998 | 15.6 |
|  | F1 | **38** | 452 | 22.1 |
|  | F2 | **50** | 283 | **40.8** |
| Thiacloprid | F0 | 6 | 1025 | 15.1 |
|  | F1 | 16 | 1425 | 27.0 |
|  | F2 | 4 | 856 | 14.6 |
| Boric acid |  | **30** | 725 | 1.9 |
| Validity criteria |  | <20 | >100 | <30 |

**
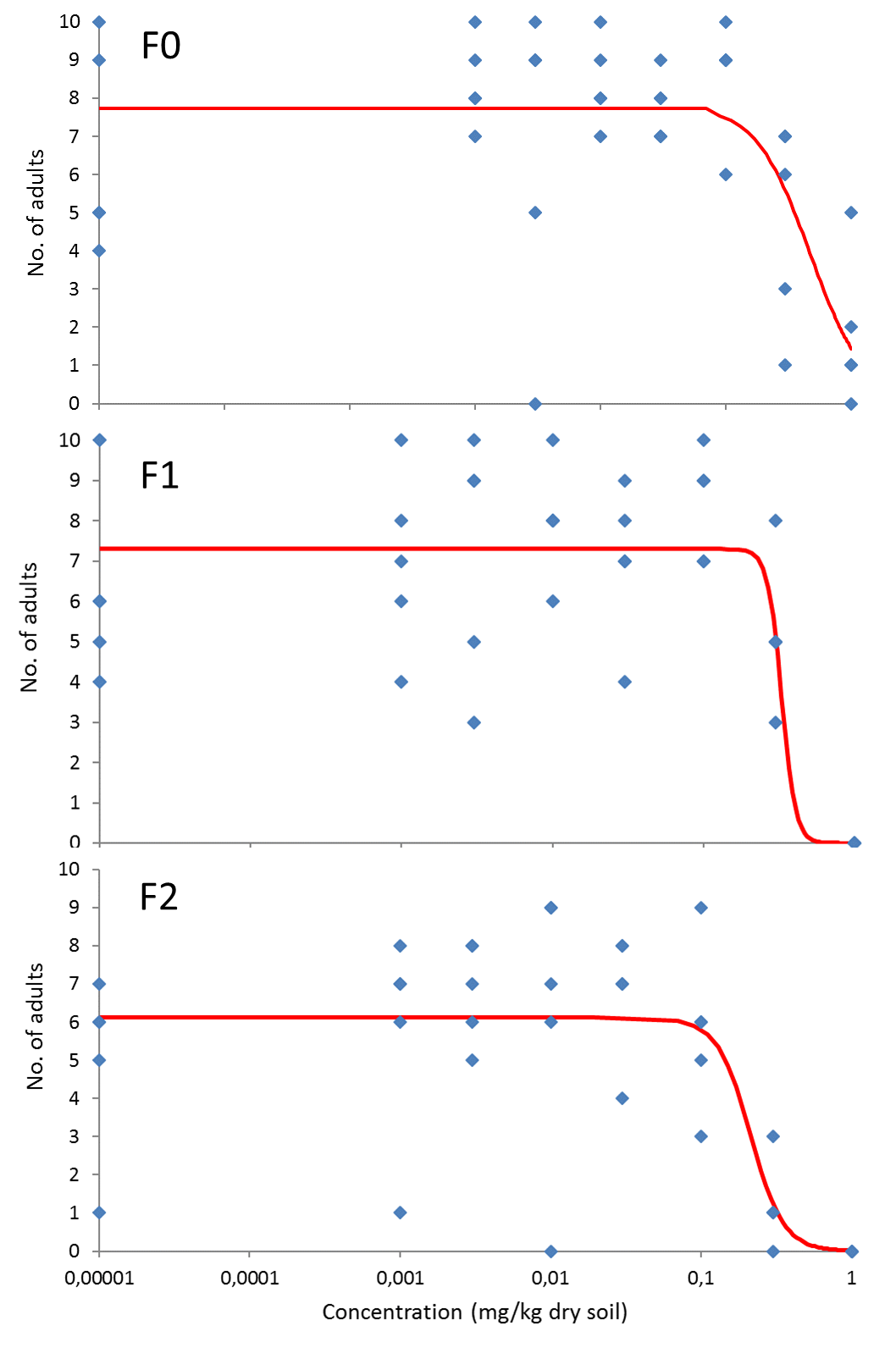
**

**Figure S1.** Dose-response relationships for the effect of imidacloprid on the survival of *Folsomia candida* exposed for three consecutive generations to LUFA 2.2 soil spiked at the start of the experiment. Concentrations are nominal values at the start of the test. The control is set at a low value of 0.00001 mg/kg dry soil. Points are measured values, lines show the fit of a logistic dose-response model to the data.

**
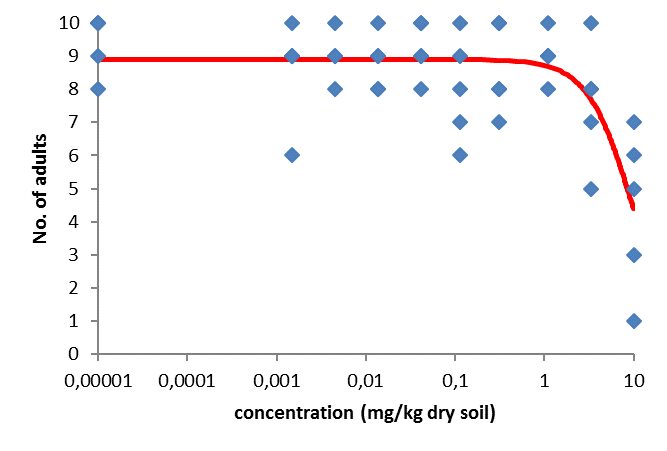
**

**Figure S2.** Dose-response relationships for the effect of thiacloprid on the survival of the first generation of *Folsomia candida* exposed in LUFA 2.2 soil spiked at the start of the experiment. Concentrations are nominal values at the start of the test. The control is set at a low value of 0.00001 mg/kg dry soil. Points are measured values, the line shows the fit of a logistic dose-response model to the data.


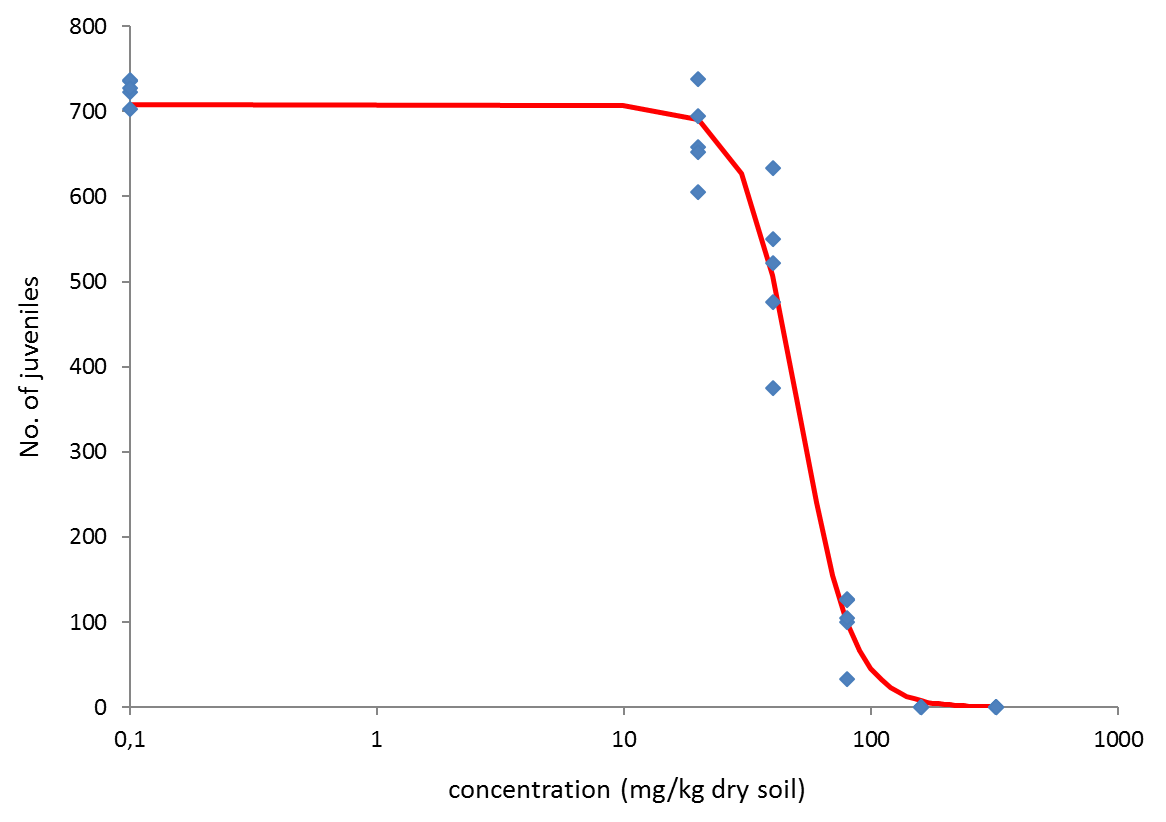


**
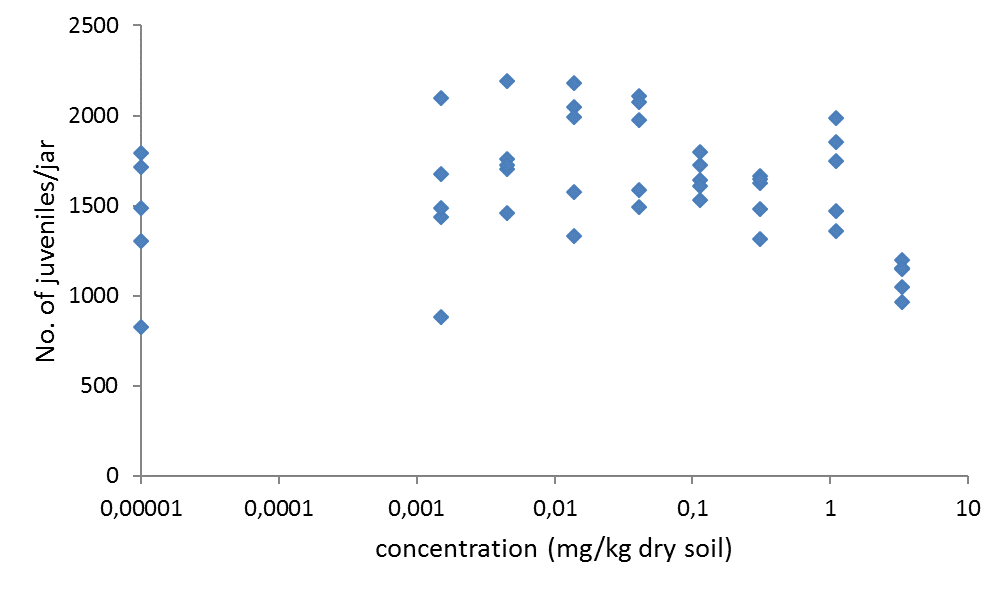

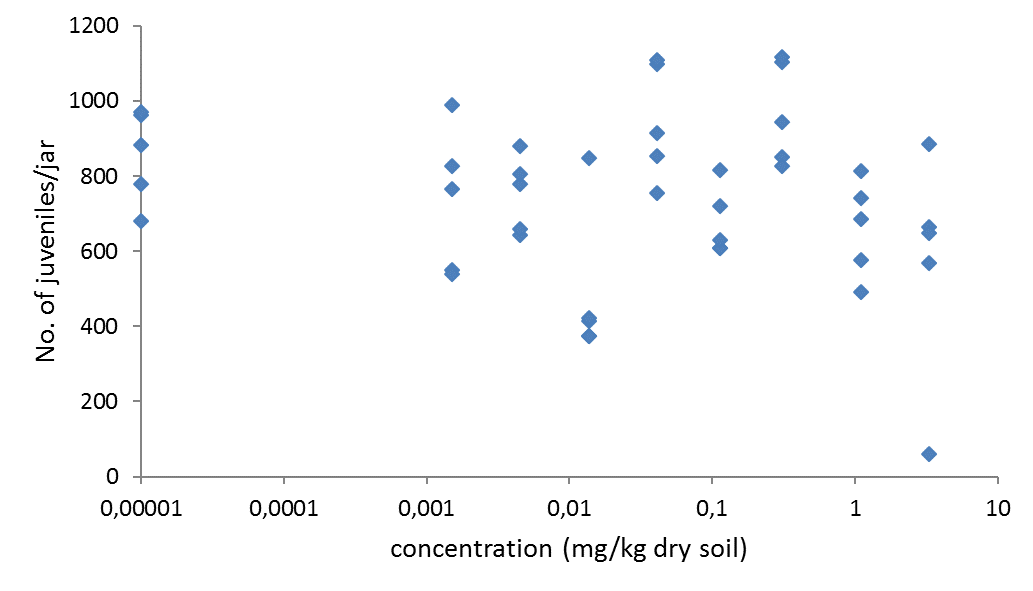
Figure S3.** Dose-response relationships for the effect of the reference compound boric acid on the reproduction of *Folsomia candida* exposed for 28 days in LUFA 2.2. Concentrations are nominal values at the start of the test. The control is set at a low value of 0.1 mg/kg dry soil. Points are measured values, the line shows the fit of a logistic dose-response model to the data.

F1

F2

**Table S3.** Overview of data on the toxicity of imidacloprid and thiacloprid to *Folsomia candida***.** All LC_50_ and EC_50_ values are in mg/kg dry soil and all tests were carried out on a 28-day exposure period. OM = organic matter.

| Chemical | LC_50_ | EC_50_ | soil type/properties | compound/formulation | Reference |
| --- | --- | --- | --- | --- | --- |
| Imidacloprid | 0.20-0.65 | 0.097-0.30 | Lufa 2.2; ~3.0% OM | Pure | De Lima e Silva et al. (submitted) |
|  | 21^#^ | >1.0* | tropical artificial soil; 10% OM (coconut husk) | Gaucho 600 FS | Alves et al. (2014) |
|  | 0.86^&^ | 0.26^&^ | artificial soil; 10% OM (sphagnum peat) | Pure | Reynolds (2008) |
|  | 2.6^#^  0.25^@^ | 0.15 | artificial soil; 10% OM (sphagnum peat) | Confidor 70 WG | Idinger (2002) |
|  | 0.21-0.44 | 0.14-0.37 | Lufa 2.2; ~3.0% OM | Pure | This study |
| Thiacloprid | 4.38 | 2.13 | artificial soil; 5% OM | Calypso 480SC | Akeju (2014) |
|  | 4.3-4.9 | 1.7-2.4 | Lufa 2.2; ~3.0% OM | Pure | De Lima e Silva et al. (submitted) |
|  | >3.33-9.0 | 1.5-3.0 | Lufa 2.2; ~3.0% OM | Pure | This study |

^#^14d LC_50_

*approx. 25% effect at highest test concentration of 1 mg/kg dry soil

^&^values recalculated from original data derived from figures included in Reynolds (2008), using a logistic dose-response model; LC_50_ and EC_50_ values reported by Reynolds based on regression analysis were 1.38 and 0.60 mg/kg, respectively

^@^recalculated from the data derived from Figure 1 in Idinger (2002) using the Trimmed Spearman Karber method (Hamilton et al. 1977/1978)

**References**

Alves PRL, Cardoso EJBN, Martines AM, Sousa JP, Pasini A (2014) Seed dressing pesticides on springtails in two ecotoxicological laboratory tests. Ecotoxicol Environ Saf 105:65-71. doi:10.1016/j.ecoenv.2014.04.010

Akeyu TO (2014) Assessment of the effects of the neonicotinoids thiacloprid and acetamiprid on soil fauna. MSc thesis, Faculty of Science and Technology, University of Coimbra, Portugal.

De Lima e Silva C, Brennan N, Commandeur D, Verweij RA, Van Gestel CAM () Comparative toxicity of imidacloprid and thiacloprid to different species of soil invertebrates. Submitted.

Hamilton MA, Russo RC Thurston RV (1977/78) Trimmed Spearman-Karber method for estimating median lethal concentrations in toxicity bioassays. Environ Sci Technol 11:714-719 (Correction: Environ Sci Technol (1978) 12:417). doi: 10.1021/es60130a004

Idinger J (2002) Laboratory studies to detect effects of selected plant protection products on *Folsomia candida* (Collembola: lsotomidae). J Plant Dis Prot 109:512-529.

ISO (2009) Soil quality - Inhibition of reproduction of Collembola (*Folsomia candida*) by soil contaminants. ISO-11267: International Organisation for Standardisation, Geneva.

OECD (2009) Guidelines for the testing of Chemicals No. 232. Collembolan reproduction test in soil. Organisation for Economic Co-operation and Development, Paris

Reynolds WN (2008) Imidacloprid insecticide treatments for Hemlock Woolly Adelgid, *Adelges tsugae* Annand (Hemiptera: Adelgidae), Affect a non-target soil arthropod community surrounding Eastern Hemlock, *Tsuga canadensis* (L.) Carriere. Master's Thesis, University of Tennessee.
